# Inverse dynamics modelling of upper-limb tremor, with cross-correlation analysis

**DOI:** 10.1049/htl.2013.0030

**Published:** 2014-05-27

**Authors:** Laurence P. Ketteringham, David G. Western, Simon A. Neild, Richard A. Hyde, Rosie J.S. Jones, Angela M. Davies-Smith

**Affiliations:** 1 Department of Mechanical Engineering, University of Bristol, Bristol, BS8 1TR, UK; 2 MS Research Unit, Bristol & Avon Multiple Sclerosis (BrAMS) Centre, Frenchay Hospital, Bristol, BS16 1LE, UK

**Keywords:** biomechanics, torque, diseases, patient treatment, inverse dynamics modelling, upper limb tremor, cross-correlation analysis, joint torques, limb motion, inertial properties, intention tremor, multiple sclerosis, body segments, patient treatment

## Abstract

A method to characterise upper-limb tremor using inverse dynamics modelling in combination with cross-correlation analyses is presented. A 15 degree-of-freedom inverse dynamics model is used to estimate the joint torques required to produce the measured limb motion, given a set of estimated inertial properties for the body segments. The magnitudes of the estimated torques are useful when assessing patients or evaluating possible intervention methods. The cross-correlation of the estimated joint torques is proposed to gain insight into how tremor in one limb segment interacts with tremor in another. The method is demonstrated using data from a single patient presenting intention tremor because of multiple sclerosis. It is shown that the inertial properties of the body segments can be estimated with sufficient accuracy using only the patient's height and weight as a priori knowledge, which ensures the method's practicality and transferability to clinical use. By providing a more detailed, objective characterisation of patient-specific tremor properties, the method is expected to improve the selection, design and assessment of treatment options on an individual basis.

## 1 Introduction

Upper-limb tremor inhibits the activities of daily living, and is a common symptom of various neuropathies, including multiple sclerosis (MS) and Parkinson's. MS is the most common disabling neurological condition that affects young adults, with the onset of symptoms typically between 20 and 45 years of age [1]. Accelerometry has been proposed as a means to improve the precision and objectivity of tremor assessment, which should improve the evaluation and development of new treatment options [2]. Uncertainty persists as to how such kinematic data translates to a measure of the patient's functional ability [3]. Electromyography (EMG) recordings can be used to grant insight into temporal patterns of muscle activation, thereby adding depth to the physiological assessment. However, without patient-specific training data, EMG data cannot be reliably translated into muscle forces, and thus cannot expose interactions between torques in neighbouring joints. This Letter describes a method with potential to characterise upper-limb tremor by quantifying the joint associated torques, thereby adding a new physiological dimension to quantitative tremor assessment.

The ultimate goals of the research are two-fold. The first is to provide a tool for clinicians that supports assessment of patients for different treatment options. The second is to support development of devices [4, 5] to reduce tremor.

## 2 Movement disorders and tremor in MS

The damage that MS inflicts on the central nervous system causes a variety of symptoms, including tremor. Tremor can be severely disabling and treatment options are limited [6]. Those with severe tremor can find their activities of daily living severely disrupted or impossible to complete without help. Tremor is estimated to affect 75% of people diagnosed with MS [6], with 38% being classed as moderate or severe cases [7]. The tremor motion is a complex movement disorder [6], often including dysmetria (a tendency to overshoot or undershoot targets) and other ataxic (un-coordinated) features.

Quantifying joint torques may provide some insight into the source of tremor. Deuschl *et al.* [8] describe tremor as having four main sources: mechanical oscillations, reflexes of the central nervous system (CNS), central oscillations in the CNS, and malfunctions in the feedforward control systems of the CNS, especially the cerebellum [6, 9].

Wrist, elbow and shoulder tremor frequencies are primarily in 3–5 Hz range [10]. Visual feedback and an increased requirement for precision in movements have both been observed as features that increase intention tremor amplitude [6, 9]. Prochazka and Trend [11] found that lower frequency tremors, such as those associated with MS, are likely to be further increased by the stretch reflex arcs linked to the muscles that actuate the elbow joint. In addition, the tremor characteristics can depend on limb positions and trajectories [6, 9]. Hence clinical indicators of tremor type and severity must also be able to record the full upper-limb motion and not just movement around a single joint.

Clinical assessments of tremor patients typically incorporate an assessment of muscle strength throughout the available range of movement (e.g. MRC scale for muscle power) and any resistance to passive movement, indicating that joint torques are indeed of clinical interest to the condition. These parameters are typically assessed by palpation or by observation of the subject's ability to resist gravity. These approaches are highly subjective and arguably imprecise. Furthermore, they can assess only the general condition of the joints and not their contribution to the tremor. To determine the role of the joints in an instance of tremor, it is essential that the measurement technique has minimal influence on the movement, for the reasons discussed in the preceding paragraph. Thus palpation is not suitable and observing resistance to gravity is limiting. Joint torque estimates derived from minimally intrusive motion capture technologies can potentially overcome these limitations.

Patients with MS are also prone to fatigue, and so the energy expenditure associated with different movements is of great clinical interest. Joint torque estimation by inverse dynamics modelling may provide a convenient means of estimating energy expenditure. Riener and Straube [12] used this approach to demonstrate that the power requirements of a subject with mild cerebellar ataxia were distinctly greater than those of a set of control subjects carrying out the same movement. In this Letter, we consider the application of inverse dynamics specifically to tremor, and propose a method for extracting clinically relevant information from the resulting data based on cross-correlations.

## 3 Patient measurements

**Figure 1:**
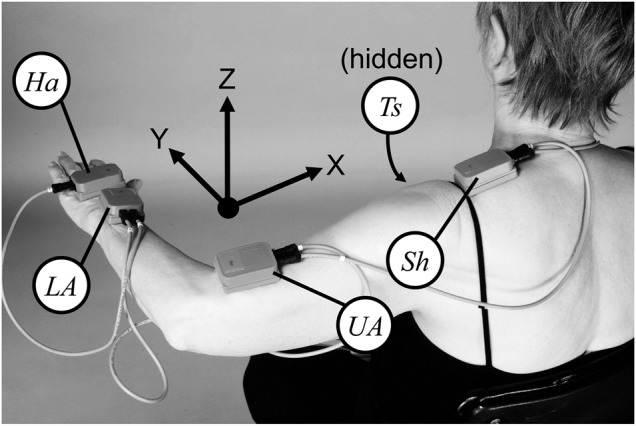
Sensor positions Torso sensor (Ts), positioned on the sternum, is obscured from view

Measurements were made using an Xbus kit (Xsens Technologies, P.O. Box 559, 7500 AN Enschede, The Netherlands), comprising five MTx sensors and an Xbus master, which was used to measure the orientations of five body segments simultaneously and transmit data wirelessly to a nearby laptop. The sensor locations are shown in Fig. 1. The orientations of the sensors attached to the torso, shoulder, upper arm, lower arm and hand were recorded at 50 Hz during the movement tests. Each sensor internally measured three orthogonal axes each of accelerometer, gyroscope and magnetometer data, and fused the data into three-dimensional orientations using proprietary algorithms. A calibration exercise was performed against a camera-based system (Qualisys) to confirm that the Xbus measurement system is able to measure both postural position and tremor motion in 3–5 Hz range. In addition, fundamental analysis of accuracy achievable from gyros and accelerometers has previously been reported [13].

During recording, the patient performed a series of finger-to-nose and reach-retrieve tests. Data logging, display and analysis software was written in MATLAB^®^ and interfaced with the libraries supplied with the Xbus kit. The movement tests, measured dimensions, sensor placements, calibration, software and analyses are described fully in [14].

## 4 Dynamic modelling

**Figure 2:**
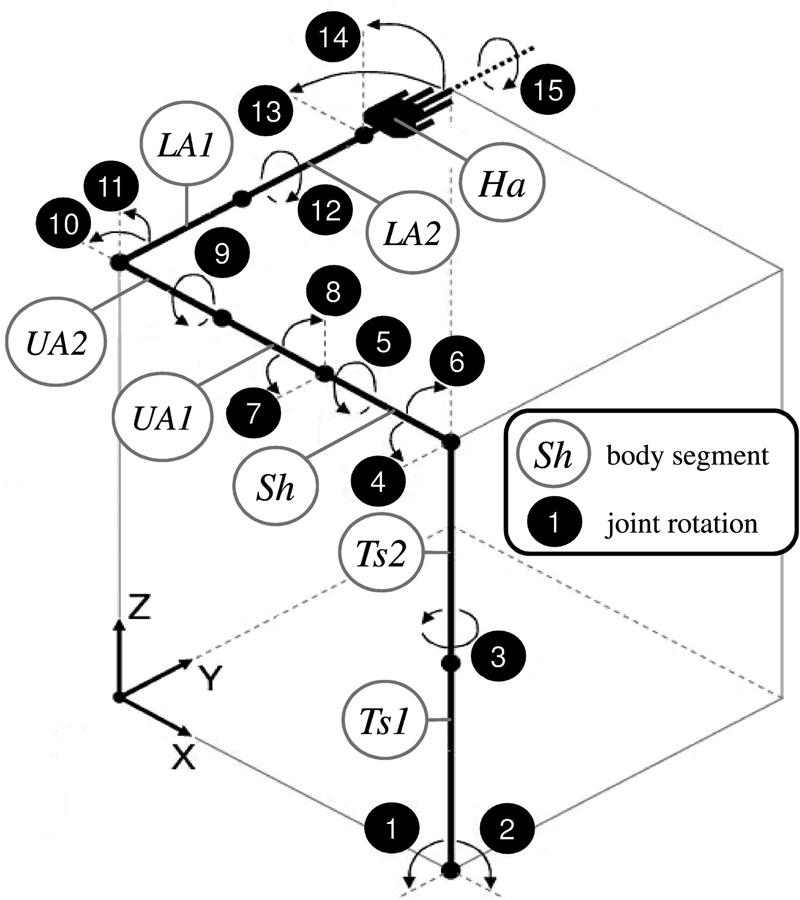
Division of body segments and joint rotation definition Ts = torso; Sh = shoulder girdle (clavicle/scapula); UA = upper arm; LA = lower arm; and Ha = hand

A 15 degree-of-freedom inverse dynamics model of the body segments and their movements was developed using the MATLAB SimMechanics™ toolbox. This model rotates body-segment masses about a set of revolute joints according to measured orientations, and allows estimation of the net torques required to perform these movements. The masses and the joint axis rotations are shown in Fig. 2 for the left arm.

The torso, upper arm and lower arm were each divided into two separate, even masses as indicated in Fig. 2, with the axial rotation occurring halfway along each body segment, between the two masses. This simplified system represented the rotation of the solid bony structures and the tissues that cover them; the flexible tissues over the bones experience little axial rotation at the proximal ends of these segments, but progressively more of the rotation of the solid bone structure beneath them is transferred to the tissues above towards the distal ends. Dempster and Gaughran [15] provide mean adult body segment lengths, masses and centres of mass relative to an individual's height and weight. Using these data, the appropriate parameters were calculated from the patient's height and weight and were attributed to rigid bodies between the joint rotations in the SimMechanics model. Errors arising from the uncertainty in these estimated parameters are assessed in Section 6.

The rigid body segments and joint axis rotations are numbered, in Fig. 2 and in the list below, from the base of the torso to the hand. Positive rotation directions are shown by the arrows in Fig. 2. These follow the rotation directions for the world axis that each joint axis is aligned with when in the zero-rotation position. Each joint axis rotation is labelled as per the world axis (*X*, *Y*, *Z*) that it is aligned with when in this position, with a subscript that is an abbreviation of the segment that it rotates.

*X*_Ts_: torso leaning backwards and forwards
*Y*_Ts_: torso leaning right and left
*Z*_Ts_: torso axial twist
*Z*_Sh_: clavicle/scapula protraction/retraction
*X*_Sh_: clavicle/scapula axial rotation
*Y*_Sh_: clavicle/scapula elevation/depression
*Z*_UA_: upper arm transverse flexion/extension
*Y*_UA_: upper arm abduction/adduction
*X*_UA_: upper arm external/internal (axial) rotation
*Z*_LA_: elbow flexion/extension
*X*_LA_: lower arm elevation/depression
*Y*_LA_: lower arm pronation/supination
*Z*_Ha_: wrist adduction/abduction
*X*_Ha_: wrist flexion/extension
*Y*_Ha_: wrist axial rotation

Some rotations can be considered minimal, namely clavicle/scapula axial rotation *X*_Sh_, lower arm elevation/depression by rotation of the elbow joint *X*_LA_ and wrist axial rotation *Y*_Ha_. However, excluding them will cause the modelled arm movement to deviate from the overall movement measured by the sensors. These small rotations are because of joint flexibility and also misalignments of the arm with the ideal zero rotation position during the calibration period.

## 5 Torque estimation

Inverse dynamics can be used to determine the torques required to produce the measured motion. For this a consistent set of kinematic data for motion at each joint is required. The angular rates and accelerations were derived from the joint angles by first applying splines to the measured data, and then calculating derivatives. The first five seconds of recorded data was used for sensor and model initialisation only. The inverse dynamics model was then simulated using the kinematic data as inputs, and the calculated joint torques were saved.

To perform frequency-based analysis of the estimated joint torques, the torque data were up-sampled from 50 to 500 Hz, this allowing a better resolution of the estimated phase differences between torques around different joints. To extract the tremor from the lower-frequency intended movements and higher-frequency noise (because of the differentiation process), the torque data was high-pass and low-pass filtered. The high-pass filter was a second-order Butterworth with cut-off at 2 Hz, and the low-pass a fifth-order Butterworth with an 8 Hz cut-off. These cut-off frequencies were the frequencies at which there was 50% attenuation of the input data, as the filter was applied twice, in both the forward and reverse directions, so as to preserve phase information.

**Figure 3:**
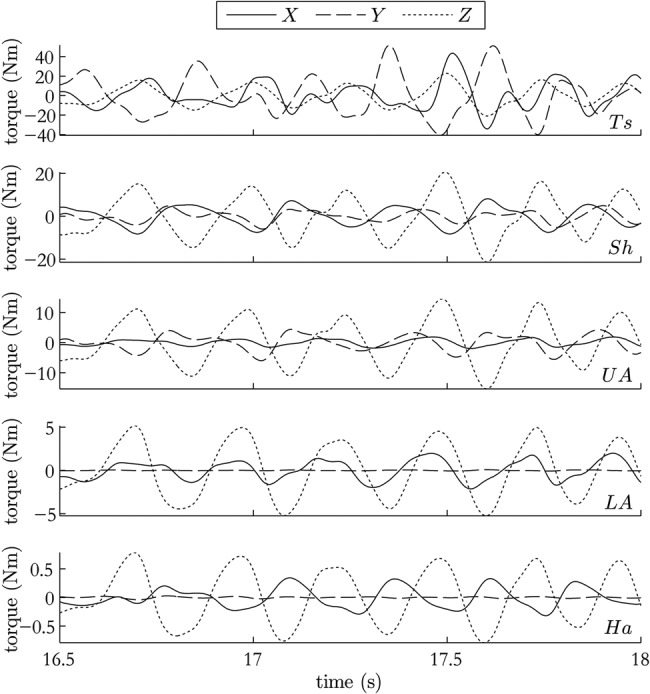
Estimated joint torques during period of high tremor in latter stages of a reaching movement

Fig. 3 shows estimated torques for a patient with MS tremor reaching towards a ball on a table. As expected, the proximal torques are much larger than the distal ones given the larger load moment arms and inertias experienced.

Torque estimation is useful for two purposes. Firstly, it can be used to inform the design of mechanical interventions where the tremor torque must be matched by an external mechanical device. Secondly it gives clinicians another way to quantify tremor severity for each joint degree-of-freedom. Purely visual assessment of individual joints’ roles in tremor is difficult because any observed rotation of a limb segment results from the combination of a net torque and a net reaction (‘bone-on-bone’) force at the joint. The linear reaction force can constitute a torque about the segment's centre of gravity, but does not reflect muscle activity (with the exception of any balanced co-contraction). The aim of inverse dynamics modelling is to isolate these influences from the net torques, which are the net contribution of muscle activity and joint impedance to the movement. Identifying these torques may help clinicians understand the source of tremor in a given patient, the causes of which can be many.

## 6 Validation of torque estimates

To isolate the joint torques, the inertial properties of each limb segment are required. Here, this is achieved by using a subject's height and weight alone to calculate these inertial properties. This approach significantly improves the practicality of the method, but is also a potential source of error because of natural variability between subjects in the true relationship between height or weight and the actual inertial parameters. To ascertain the effects of uncertainty in the estimated inertial properties of the body segments, multiple torque estimations were carried out using the same kinematic data. The length and mass of each of the five segments was set as either mean +*σ* or mean −*σ*, where *σ* is the standard error in that parameter, determined from data in [15] on the standard deviation of body segment properties among adults, relative to an individual's height and weight. A total of 2^10^ = 1024 simulations were executed to test every possible combination of the ten uncertain parameters.

For each simulation, the effect of the alternative parameters on the torque estimate for each joint axis was quantified by determining the difference between the estimated torque and that found in the benchmark run (where only the mean parameters were used). The root-mean-squared value (RMS) of this error series was calculated as a percentage of the RMS of the corresponding torque series in the benchmark run. Results are shown in Table 1.

**Table 1:** Mean value (and max in brackets), over the 1024 simulations, of the RMS of torque error as a percentage of the RMS of the benchmark torque series, for each degree-of-freedom

Segment	*X*, %	*Y*, %	Z - %
hand	3.6 (19.0)	1.9 (19.0)	2.8 (19.0)
lower arm	4.0 (12.8)	3.4 (16.6)	3.4 (13.8)
upper arm	4.0 (12.7)	5.0 (21.5)	4.1 (12.5)
shoulder	4.4 (14.9)	4.9 (20.4)	4.0 (10.7)
torso	3.6 (14.5)	3.1 (13.7)	4.1 (10.5)

A further potential source of error in our method is the spline differentiation process used to calculate angular velocities and accelerations from the orientations returned by the sensor system. Validation of this kinematic aspect of our approach was carried out by modelling a simpler system, a double pendulum with constant torque added at each of the two joints. A forward simulation of this system was used to generate a kinematic data set. Angular velocities and accelerations were calculated from the angles using the spline differentiation approach mentioned above, and these values were compared against the true values calculated directly through the forward dynamics simulation. The series were almost indistinguishable (see figure 6.11 in [14]), with maximum errors of 0.7 and 2.4% at the velocity and acceleration peaks, respectively. A more detailed description of this validation process is reported in [14].

Although the validation was not applied to true tremor kinematics, and we have not characterised the impact of inter-individual variability in tremor characteristics, the parameters of the double pendulum system were chosen so as to yield velocities and accelerations (max 10^3^°/s and 10^4^°/s^2^) that conservatively exceeded typical tremor kinematics. Hence the dominant source of error in our method is deemed to be the uncertainty in inertial parameters, rather than kinematic variability between individuals or the method of synthesising of a full kinematic data set.

## 7 Cross-correlation analysis

A way to explore how motion in one degree-of-freedom may be influencing motion in another is to look at the cross-correlation of corresponding joint torques. As the torque estimation method described removes most of the inertial effects, any significant cross-correlation will be caused by the muscle and tendon physics plus neurological control pathways. This makes it potentially easier to make inferences about the causes of an individual's tremor than if looking at just the motion itself.

To demonstrate the process by which cross-correlation might be used, the tremor data in from the reach-retrieve task was analysed. Cross-correlations of the filtered torque data were carried out on three adjacent 1.5 s epochs at the beginning of the first reaching movement. At these times, the subject was resting before movement, in the transition period from resting to movement, and moving towards the ball at the far cup. The results shown in Fig. 3 are for this third epoch. Cross-correlations of each of the 15 joint axes with each of the other 14 joint axes were calculated and normalised to lie between 0 (no correlation) and 1 (identical series).

**Figure 4:**
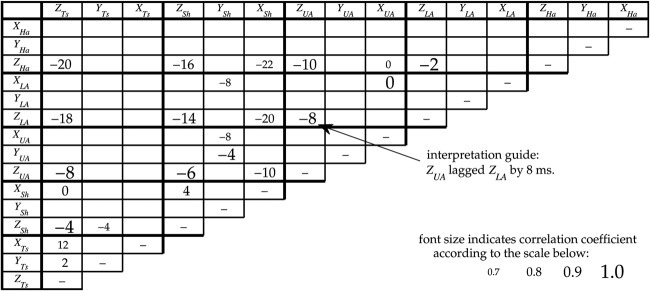
Cross-correlations during reaching Each cell shows the time difference, in milliseconds, by which the axis corresponding to that column led the axis corresponding to that row, in terms of the torque about each axis As indicated by the scale in the lower-right corner, the font size indicates the magnitude of the correlation coefficient between the two torque series at this lag For example, the Z-axis of the upper arm lagged that of the lower arm by 8 ms, with a correlation coefficient >0.9 Lags are not shown for axis pairs with a peak correlation coefficient of <0.75

Cross-correlation results are summarised in Fig. 4. Each value in the cross-correlation grid shows the time by which the torques in one joint axis led or lagged behind those in another axis, while the level of correlation that was found between the compared torque waveforms is indicated by the size and lightness of the values in the grid. Where torque waveforms were well correlated the numbers are larger and darker; where they were not well correlated, and therefore provide less reliable lead/lag values, they are shown with a smaller, paler value in the grid.

The clearest set of correlations are those between joint axes that rotate around the *Z*-axis. It can be seen that this pattern was led at the hand *Z*_Ha_, while the lower arm, upper arm, shoulder and torso lagged the hand by 2, 10, 16 and 20 ms, respectively. Although the lead/lag times varied slightly, the same pattern and high correlations were found between the *Z*-axis rotations throughout the entirety of the reaching movements. This suggests that tremor in these joints is either coupled or driven by a common input. The latter, or some combination of the two, is plausible given the well-known role of visual feedback in intention tremor [6, 9]; the *Z*-axis would all be driven in response to the same horizontal errors.

The strong correlation and indiscernible lag between *X*_UA_ and *X*_LA_ are not of clinical interest. This interaction reflects the fact that *X*_LA_ is not an anatomical degree-of-freedom; there is a rigid mechanical coupling between axial rotation of the upper arm and rotation of the lower arm about the same axis.

The observation that tremor torques, at least for this patient, are strongly linked across multiple joint axes is encouraging for the development of devices to alleviate intention tremor. It is conceivable that a device that suppresses tremor in only a subset of these axes would lead to a small reduction in the position errors that are visually fed back. This reduction in the error signal might then lead to a reduced tremor magnitude across all axes, thus allowing the system to converge towards a less tremulous state. A similar reduction could be expected for aspects of the tremor that are driven by stretch reflex arcs around the joints. In this sense, we anticipate that our method could be useful as a means of targeting a minimal set of joints to achieve tremor reduction in an individual. This could be an important contribution to the development of mechanical tremor suppression devices, since these have previously been hampered by excessive bulk and aesthetic considerations (e.g. [4]).

Given that the results presented here are for a single patient, no conclusions can be drawn at this stage as to the more general utility of the cross-correlation analysis to support patient assessment. Clinical trials are about to take place with a larger patient cohort which will help understand relevance to clinical assessment, including the ability to predict when specific interventions are likely to be beneficial.

## 8 Conclusions

A clinically convenient approach to using inverse dynamics to unobtrusively estimate tremor joint torques has been demonstrated and validated with regard to numerical considerations. The modelling approach is kept simple so that only the patient's height and weight are required to estimate the body segment inertial properties. This makes the approach practical in a clinical setting by reducing the a priori information required. The impact of this simplification on the accuracy of the torque estimates was characterised and deemed to be acceptable, given the practical advantages. Further studies with a larger patient group will allow a more detailed characterisation of the method's reliability.

It has been argued that estimating torques enables a more detailed characterisation of tremor movements than would be possible from a purely kinematic analysis. Joint torque correlations can be used to infer causal relationships between tremors in individual joint axes, an approach which was shown to be robust to uncertainties in the inertial properties of individual limb segments. The insights yielded by the technique may help clinicians to infer the neuromuscular origins of tremor on a patient-specific basis, which is a goal for the further development of tremor treatment.

The specific results presented in this Letter are being used to support sizing of a mechanical intervention for tremor. From the results, there is some evidence that tremor reduction using mechanical intervention at a distal joint could result in corresponding reduction in a more proximal joint. A forthcoming clinical trial involving more patients is expected to throw more light on this.
